# Examination of effects of GSK3β phosphorylation, β-catenin phosphorylation, and β-catenin degradation on kinetics of Wnt signaling pathway using computational method

**DOI:** 10.1186/1742-4682-6-13

**Published:** 2009-07-22

**Authors:** Ying-Chieh Sun

**Affiliations:** 1 Department of Chemistry, National Taiwan Normal University, 88, TingChow Road Section 4, Taipei 116, Taiwan

## Abstract

**Background:**

Recent experiments have explored effects of activities of kinases other than the well-studied GSK3β, in wnt pathway signaling, particularly at the level of β-catenin. It has also been found that the kinase PKA attenuates β-catenin degradation. However, the effects of these kinases on the level and degradation of β-catenin and the resulting downstream transcription activity remain to be clarified. Furthermore, the effect of GSK3β phosphorylation on the β-catenin level has not been examined computationally. In the present study, the effects of phosphorylation of GSK3β and of phosphorylations and degradation of β-catenin on the kinetics of the wnt signaling pathway were examined computationally.

**Methods:**

The well-known computational Lee-Heinrich kinetic model of the wnt pathway was modified to include these effects. The rate laws of reactions in the modified model were solved numerically to examine these effects on β-catenin level.

**Results:**

The computations showed that the β-catenin level is almost linearly proportional to the phosphorylation activity of GSK3β. The dependence of β-catenin level on the phosphorylation and degradation of free β-catenin and downstream TCF activity can be analyzed with an approximate, simple function of kinetic parameters for added reaction steps associated with effects examined, rationalizing the experimental results.

**Conclusion:**

The phosphorylations of β-catenin by kinases other than GSK3β involve free unphorphorylated β-catenin rather than GSK3β-phosphorylated β-catenin*. In order to account for the observed enhancement of TCF activity, the β-catenin dephosphorylation step is essential, and the kinetic parameters of β-catenin phosphorylation and degradation need to meet a condition described in the main text. These findings should be useful for future experiments.

## Background

The Wnt/β-catenin signaling pathway (named wnt pathway hereafter for simplicity) plays a significant role in cell proliferation, differentiation, and apoptosis. These have implications for aspects of cell development, stem cells and cancer [1]. Many characteristics of this pathway and its role in cell signaling have been revealed in experimental studies (for review, see for example [1], and references therein and the literature listed at ). Briefly, wnt signaling enhances the level of the output signal protein, unphosphorylated β-catenin, which then binds with TCF to induce associated gene expression in the nucleus. At steady state (SS), the level of β-catenin is balanced by its synthesis and degradation. The so-called destruction cycle is a major mechanism of degradation, in which phosphorylation of β-catenin by GSK3b is a key step [2]. When the wnt signal acts on cell, wnt recruits several proteins to attenuate the reaction rate of this key step, slowing down the degradation. Therefore, β-catenin accumulates, enhancing the level of TCF/β-catenin complex and the resulting associated gene expression.

Recent advances have further illustrate how phosphorylation and dephosphorylation of major components in the wnt pathway affect the stability of β-catenin and its TCF transcription activity [3-7]. It has been found that LRP6 phosphorylates GSK3β and regulates β-catenin independently of the axin pathway [3]. PKA phosphorylates GSK3β and affects the β-catenin level in Saos-2 cells [4]. β-catenin is also phosphorylated by AKT at Ser552, promoting TCF activity [4]. Furthermore, PKA phosphorylates Ser552 and Ser675 of β-catenin but this does not affect the β-catenin level in COS7 cells [5]. Moreover, CK1 phosphorylates not only axin and APC but also GSK3β and β-catenin [6]. A newly identified component, PP1, dephosphorylates axin [6]. In addition, phosphorylation of β-catenin at Ser675 by PKA attenuates β-catenin degradation, stabilizing β-catenin and enhancing TCF activity in the cells investigated [7]. In aspects of pathology, β-catenin has a role in carcinogenesis although the extent of its effect varies among cancers [8]. For example, the effect on colorectal cancer is more significant than on lung cancer. In a recent study [9], a derivative of celecoxib, derivatives of which have been extensively examined for anti-cancer treatment, was found to have potential for treating lung cancer. Proteomics examination showed that PKA activity has a significant effect on the wnt signaling pathway and in differentiating lung from normal cells.

**Figure 1:**
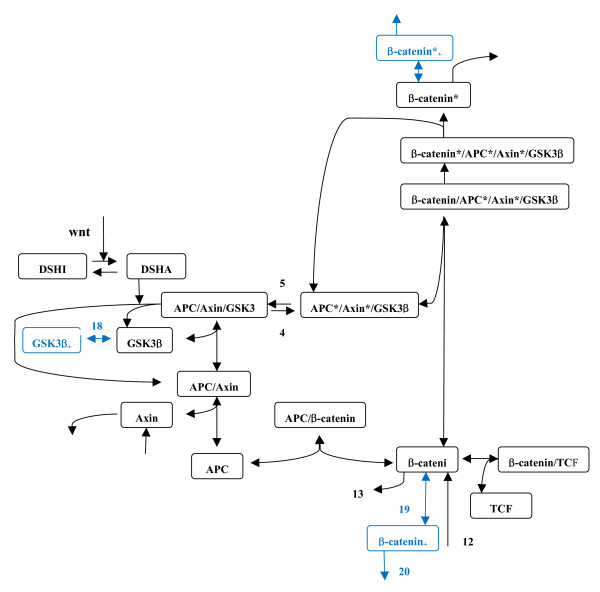
**Schematic diagram of the modified Lee-Heinrich model of wnt pathway**. The modified model includes GSK3β phosphorylation, β-catenin non-GSK3β phosphorylation and degradtion denoted by reaction steps 18, 19, and 20, respectively, shown in blue. The reaction rate constants of these reaction steps are denoted k_G_/k_G_' (forward/backward reaction), k_β_/k_β_', and k_βdeg_, respectively. The added reaction steps at β-catenin* shown in the upper part of diagram are similar to the added reaction steps at β-catenin, denoted 19 and 20 (see text for detailed description). Numbering of other reaction steps is the same as in the original Lee-Heinrich model [2].

In addition to experimental studies, computational studies have also aided understanding of the dynamical behaviors of this pathway and how it interacts with other pathways [2,10-13]. The wnt pathway is one of the computationally best-studied signaling pathways [14-16]. The kinetics of β-catenin, axin, and other proteins have been examined in the well-established Lee-Heinrich model [2], following the nomenclature in [12], which was built on the basis of experiments with Xenopus. Briefly, the Lee-Heinrich model contains a number of key protein components in the wnt pathway shown in Figure 1[2]. (Note that a kinase, casein kinase 1 (CK1), was recently implicated in the activation of β-catenin phosphorylation by GSK3β [6], and this was not included in the Lee-Heinrich model. This kinase is not directly related to the three effects examined in the present study. Therefore, it is not included here.) Binding of proteins, catalytic activities, phosphorylation reactions, etc. are described by their corresponding kinetic rate laws with the associated parameters, which were obtained from measurements or estimated [2]. Effects of wnt signaling and changes of activity of some components on the kinetics of the pathway can be investigated by solving kinetic rate law equations [2]. A number of the effects examined and the kinetics of components were in excellent agreement with experimental results [2].

In addition to the Lee-Heinrich model of the wnt pathway itself, this model has been used and extended to examine the effect of Apc mutations on the wnt signaling pathway [10], cross-talk with the ERK pathway [11], and the interaction of axin2 proteins with the wnt pathway [12]. While many characteristics of this pathway have been elucidated both experimentally and computationally, much of its role in cell signaling and the means by which it interacts with other pathways remain to be explored.

In the present study, in the light of the recent experimental studies described above [3-7], I aimed to examine the effects of GSK3β phosphorylation, β-catenin phosphorylation by kinases other than GSK3β (referred to as β-catenin non-GSK3β phosphorylation hereafter), and β-catenin degradation on the kinetics of the wnt pathway using a computational method based on the Lee-Heinrich model. These effects were not included explicitly in the Lee-Heinrich model, and to my knowledge, they have not previously been examined computationally. The present computational study should elucidate how these effects affect the wnt pathway. The reaction steps of GSK3β phosphorylation, β-catenin non-GSK3β phosphorylation, and β-catenin degradation were added to the Lee-Heinrich model (see added reaction steps in blue in Figure 1). The dependence of SS concentrations on the associated kinetic parameters of interest in these reaction steps was examined. Control coefficients of selected parameters and time courses of selected components with wnt signalling were calculated and examined as well. The method is described in Section II. Section III presents the results and discussion. Conclusions are given in the final section.

## Methods

The COPASI program [17] was used to solve kinetic rate equations numerically in order to simulate the wnt pathway. I first built the Lee-Heinrich model of the wnt pathway and reproduced the SS concentrations and several time course concentration curves, shown in Table 2 and Figure 6 in Ref. [2], respectively. The reproduced results are shown in Table 1 and the solid lines in Figure 3. The newly-added and examined effects of GSK3β and β-catenin non-GSK3β phosphorylations were assumed to involve free GSK3β and β-catenin only. An experimental study [7] showed that these two proteins are less likely to be phosphorylated in the complexes than in the free forms. Additions of these two reaction steps along with their dephophorylation steps, assigned as reaction steps 18 and 19, respectively, and degradation of β-catenin, reaction 20, are shown in blue in Figure 1. The rate laws for these reactions are the kinetic equations for standard first order reversible (steps 18 and 19) and irreversible (step 20) reactions. β-catenin non-GSK3β phosphorylation and degradation were assumed to take place independently with un-phosphorylated β-catenin and GSK3β-phosphorylated β-catenin, as shown in the lower and upper parts of Figure 1, respectively. These processes were also examined separately. The effects of these added reactions steps on the kinetics of the pathway were examined.

**Table 1:** Steady-state concentrations of selected components in the Lee-Heinrich and modified models

	Concentration (nM)		
	
component	Lee-Heinrich model	modified model	Fold change
Dsh_a_	0	0	
(APC*/axin*/GSK3β)	0.00966	0.004742	0.491
(APC/axin/GSK3β)	0.00483	0.002362	0.489
(β-catenin*/APC*/axin*/GSK3β)	0.00202	0.001991	0.986
β-catenin*	1.00	0.983341	0.983
β-catenin	25.1	50.3766	2.007
Axin	0.000493	0.000492	0.998
GSK3β	50	25	0.500
GSK3β*		25	
β-catenin_+_		5.03766	

(see text for description).

The forward/backward reaction rate constants of GSK3β and β-catenin phosphorylations are denoted k_G_/k_G_' and k_β_/k_β_', respectively. The rate constant of β-catenin degradation is denoted k_βdeg_. For simplicity, these kinetic parameters were all set equal to 1 min^-1 ^except in some cases (see Section III). The parameters of interest were then varied to examine their effects on the kinetics of the wnt pathway. Initial concentrations were values of SS concentrations used in the Lee-Heinrich model [2] with additional components, GSK3β_+_, β-catenin_+_, and β-catenin*_+_, with initial concentrations all set at zero. The superscript * denotes phosphorylation by GSK3β. Other phosphorylations are denoted by the subscript_+_. The differential equations were solved for 20000 minutes, or as long as 40000 minutes, to ensure they reached SS.

To examine how selected parameters affect the SS concentration of β-catenin in a modified model, control coefficients were calculated as well. These were defined by [2]



where β-*cat *is concentration of β-catenin and *k *is the parameter of interest. This coefficient was calculated numerically by varying the associated parameters by 1% and solving the kinetic equations over enough time to obtain SS concentrations, in order to calculate control coefficients as in [2]. Finally, the wnt signaling effect in a modified model (see below) was also examined with constant wnt signaling and transient wnt signaling separately, as in [2]. Initial concentrations in the modified model were obtained from computations for SS concentrations. Computed results are presented and discussed in the next section.

## Results and discussion

### Effects of GSK3β phosphorylation and β-catenin phosphorylations

Initially, I examined these two effects without including the β-catenin degradation step (step 20 in Figure 1) in computing β-catenin concentration, and assumed that β-catenin non-GSK3β phosphorylation involves β-catenin only (reaction step 19 in lower part of Figure 1). Computed SS concentrations of selected components and their fold changes are listed in Table 1, along with values in the original Lee-Heinrich model [2]. The concentration of GSK3β was reduced to half its original value. The values of complexes with APC and axin were also reduced to approximately half their original values while β-catenin*/APC*/axin*/GSK3β, β-catenin*, and axin remained approximately the same. The unphosphorylated β-catenin shows a twofold change and significant enhancement in its absolute concentration because of its high concentration compared with most of the other components.

**Figure 2:**
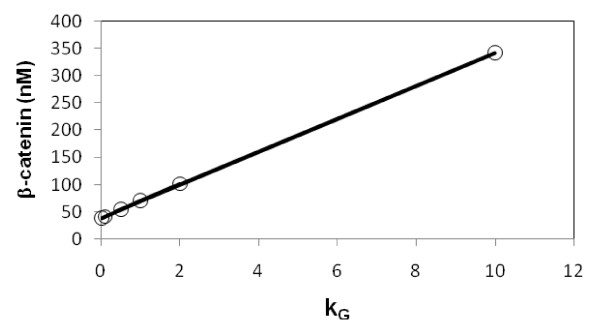
**Dependence of level of β-catenin on GSK3β phosphorylation**. Circles are computed results. The line shows that the dependence is almost linear. The other kinetic parameters of added reaction steps, k_G_' and k_β_/k_β_', were set equal to 1 and 0.1/1 min^-1^, respectively.

To examine how the kinetic parameters of the added reaction steps affect the kinetics of the wnt pathway, selected parameters were varied and the rate laws of the modified model were solved. The SS solutions were examined first. The effect of varying k_G _on the total β-catenin level and free unphosphorylated β-catenin are listed in Table 2. The higher the k_G _value, the higher the β-catenin level. This dependence qualitatively demonstrates the negative role of GSK3β in wnt/β-catenin signaling. This is because a decrease in unphosphorylated GSK3β level results in a decrease in the APC/axin/GSK3β complex. This complex is a central component of the β-catenin destruction cycle. Therefore, the level of β-catenin accumulates and increases. Experimentally, it was found that phosphorylation of GSK3β led to enhancement of the β-catenin level in HEK293-TPα cells [18] but not HEK293 cells [19]. The discrepancy is due to differences in cell type, experimental conditions, etc. The present result is consistent with the former cell type. The GSK3β/GSK3β_+ _ratio is determined by the k_G_/k_G_' ratio. The dependence of the k_G_/k_G_' ratio on β-catenin level is shown in Figure 2, a plot of ratio of β-catenin level versus k_G _that represents the strength of phosphorylation of GSK3β by other kinases. This plot shows an almost linear dependence. Because of the significant effect when k_G_/k_G_' = 1 (Table 2), this parameter set was used as a reference parameter set to analyze the effects of β-catenin non-GSK3β phosphorylation on the wnt pathway in the discussion below.

**Table 2:** Steady-state concentrations of total and unphosphorylated β-catenin (in nM) under conditions of different k_G_/k_G_' ratios

k_G_/k_G_' ratio	SS concentration of β-catenin in total	SS concentration of unphosphorylated β-catenin
10	342.1747	280.744
2	101.1289	75.8736
1	69.83185	50.3766
0.5	53.8597	37.6965
0.1	40.78498	27.5907
0.01	37.78723 34.94785^a^	25.3219 25.0699^a^

^a ^values of original Lee-Heinrich model.

In addition to the effects of GSK3β phosphorylation, the effect of β-catenin non-GSK3β phosphorylation was examined. I first examined this effect in free unphosphorylated β-catenin (lower part of Figure 1). Analysis of pathway fluxes showed that no SS concentrations of components are affected by the k_β_/k_β_' ratio, excepting concentrations of β-catenin_+_. This was confirmed by computations with several values of k_β_/k_β_'. This is because β-catenin is one of two components, in addition to axin, that have flux turnovers in the pathway. Because of this, addition of reaction step 19 to the Lee-Heinrich model does not change the flux-in or flux-out at SS. This is in contrast to phosphorylation of GSK3β, when the k_G_/k_G_' ratio does affect the concentrations of components at SS. This is because the total amount of GSK3β is conserved and has no turnover. Therefore, reaction step 18 decreases the level of unphosphorylated GSK3β and increases the level of β-catenin. This shows that phosphorylation of GSK3β has a significant effect on the β-catenin, but β-catenin non-GSK3b phosphorylation has no significant effect on concentrations of components in the destruction cycle in this model. In addition to this calculation examining the effect of non-GSK3β phosphorylation of free unphosphorylated β-catenin, I also considered this effect as taking place separately at the β-catenin* shown in the upper part of Figure 1. Because the pattern of reaction steps is similar, similar results for the dependence of β-catenin*_+ _level on the kinetic parameter k_β_/k_β_' were expected and obtained in the computations.

In addition to SS concentrations, control coefficients of selected parameters were also calculated to understand how these parameters affect SS concentrations. Because parameter k_G _in reaction step 18 has a significant effect on the level of β-catenin, I calculated the control coefficients of this parameter along with several selected parameters, k_4_, k_5_, and v_12_. The results are listed in Table 3. The control coefficient of k_G _was 0.449, close to half of the control coefficient of GSK3β, which is 0.89, in the original Lee-Heinrich model. Other control coefficients do not differ significantly from the original Lee-Heinrich model. These results showed that addition of reaction steps 18 and 19 in the modified model does not significantly change the control coefficients of β-catenin of rate constants k_4 _and k_5 _and the influx rate, v_12_. It does significantly change in the case of k_G _in reaction step 18.

**Table 3:** Control coefficients of β-catenin (concentration in total) with respect to selected kinetic parameters.

	Control coefficient for β-catenin	
	
Kinetic parameter	Lee-Heinrich model	modified model
k_4_	-0.89	-0.89786
k_5_	0.89	0.906281
v_12_	0.929	0.949923
k_G_		0.453225

(see text for description)

### Effect of wnt signal

In examining the wnt signaling effect, computations for the present modified model were carried out similarly to those in the previous work [2]. Computations with constant wnt signaling of W = 1 and with transient signaling were carried out separately. For constant signaling, the calculated SS concentrations of selected components when W = 1 are listed in right part of Table 4. Addition of reaction steps 18 and 19 does not significantly affect the concentration of Dsha. A noticeable change is β-catenin, which is again about twice the value obtained in the original Lee-Heinrich model. This is similar to the case without wnt signaling (W = 0). This shows that the effect of constant wnt signaling in SS in the present modified model with (W = 1) is similar to the original Lee-Heinrich model.

**Table 4:** Steady-state concentrations of selected components without and with constant wnt signaling in the Lee-Heinrich and the present modified model

	**W = 0**		**W = 1**	
		
**Components**	**Lee-Heinrich model**	**Modified model**	**Lee-Heinrich model**	**Modified model**
Dsh_a_	0	0	90.9091	90.9091
(APC*/axin*/GSK3β)	0.00966	0.004742	0.001461	0.000656
(APC/axin/GSK3β)	0.00483	0.002362	0.000728	0.000327
(β-catenin*/APC*/axin*/GSK3β)	0.00202	0.001991	0.001862	0.001672
β-catenin*	1.00	0.983341	0.920076	0.825947
β-catenin	25.1	50.3766	153.028	305.759
Axin	0.000493	0.000492	0.000492	0.000492
GSK3β	50	25	50	25
GSK3β_+_		25		25
β-catenin_+_		5.03766		30.5759

(see text for description).

**Figure 3:**
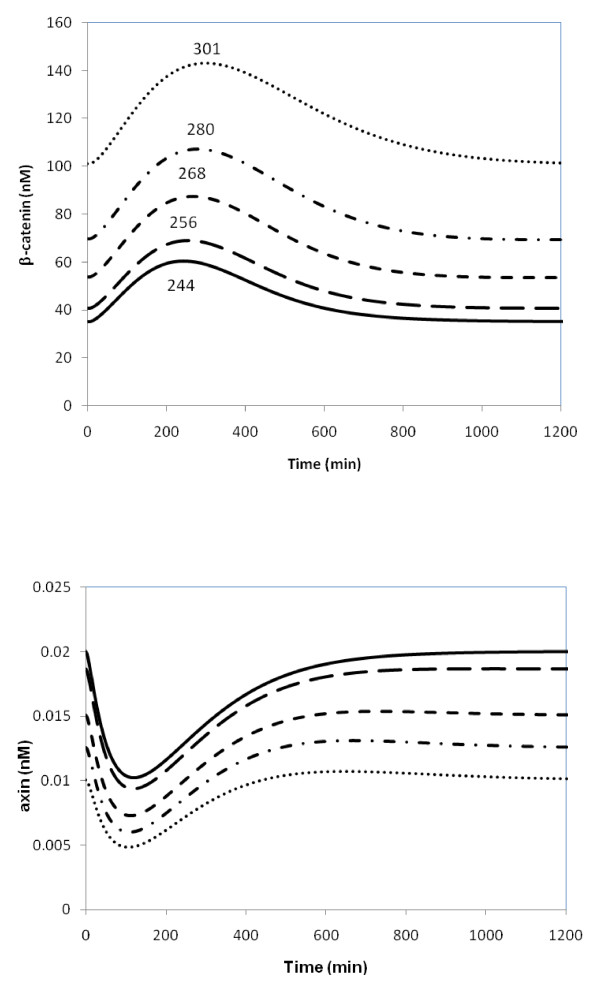
**Time course of total concentrations of β-catenin and axin with transient wnt signaling**. Solid line: the original Lee-Heinrich model. Other lines: k_G _parameter was varied. Long dashed line: k_G _= 0.1 min^-1^. Short dashed line: k_G _= 0.5 min^-1^. Dashed-dotted line: k_G _= 1 min^-1^. Dotted line: k_G _= 2 min^-1^. The other kinetic parameters of added reaction steps, k_G_' and k_β_/k_β_', were set equal to 1 and 0.1/1 min^-1^, respectively. The time (in minutes) to maximum β-catenin level is marked for each line.

Because wnt signaling interacts with the destruction cycle directly through the the APC/axin/GSK3β complex and because GSK3β has a significant effect on the β-catenin level, I examined how varying k_G _affected the time course of β-catenin and axin. As in [2], I used an exponential decay wnt signaling with a decay rate of 20 minutes. The time course of total β-catenin concentration is shown in Figure 3. The response of axin is similar to that in the original Lee-Heinrich model, but the response of β-catenin lags behind slightly.

### Effect of β-catenin degradation

**Figure 4:**
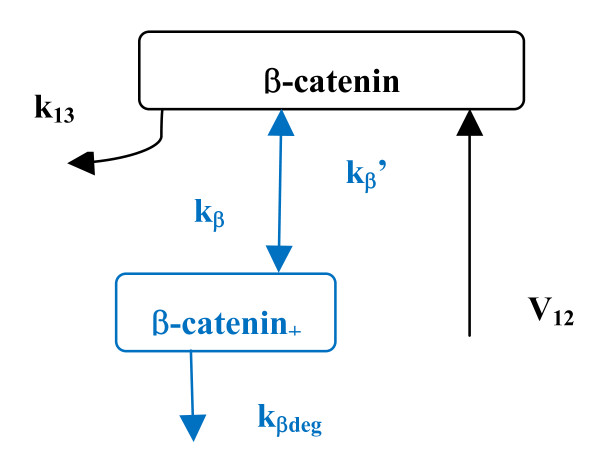
**Schematic diagram of simplified model of β-catenin non-GSK3β phosphorylation and degradation reaction steps**. The reaction rate constants of these reaction steps are denoted k_β_/k_β_' (forward/backward reaction) and k_βdeg_, respectively (see text for detailed description). Numbering of reaction steps 12 and 13 is the same as in the original Lee-Heinrich model [2].

**Figure 5:**
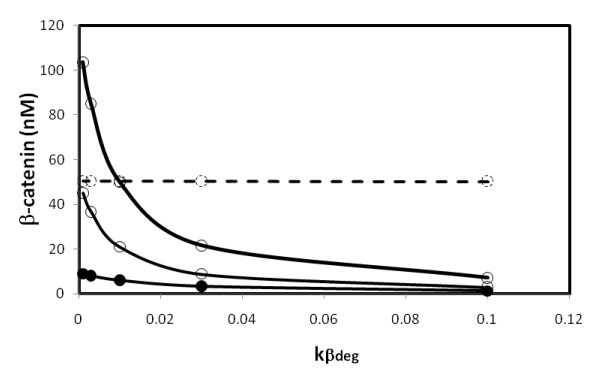
**Dependence of level of β-catenin on β-catenin_+ _degradation**. Thick line with open circle: total β-catenin level. Thin line with open circle: unphosphorylated β-catenin level. Kinases such as PKA inhibit β-catenin degradation. Higher PKA activity corresponds to lower degradation rate constant *k*_β*deg*_, giving higher β-catenin level. Other kinetic parameters of added reaction steps, k_G_/k_G_' and k_β_/k_β_', were set at 1/1 and 1/1 min^-1^, respectively. Thin line with solid circle: TCF/β-catenin level. Dashed line: the results when k_β _= k_13_. Unphosphorylated β-catenin level is shown. The result for the total β-catenin level is similar, not shown here (see text for detailed description).

Similar to the examinations of the effect of β-catenin non-GSK3β phosphorylation described above, degradation of β-catenin may involve free unphosphorylated β-catenin or β-catenin* as shown in Figure 1. To examine this effect, separate computations were carried out for degradation taking place in these two components. The behaviors of SS concentrations were qualitatively similar for these two cases when the associated parameters were varied, although the former case involved more reaction steps and the latter was more 'isolated' from the destruction cycle. Qualitative behaviors of SS concentrations can be understood by a simplified kinetic model shown in Figure 4. In this simplified model, the SS concentration of unphosphorylated β-catenin, and the sum of β-catenin and β-catenin_+_, can be expressed by

(1)

and

(2)

respectively. The computations for the added reaction steps taking place at β-catenin*_+ _(upper part of Figure 1) showed that the dependence of β-catenin level on the kinetic parameter is qualitatively similar to the simple functions shown above. Similar results were obtained with computations for the added reaction steps at the β-catenin site (lower part of Figure 1). To elucidate the downstream TCF activity, only added reaction steps on free unphosphorylated β-catenin shown in the lower part of Figure 1 are considered here. This is because these reaction steps can alter the concentration of free unphosphorylated β-catenin, which in turn will interact with TCF to induce downstream transcription. To account for observed enhancement of TCF activity induced by increased kinases such as PKA, which phosphorylates β-catenin and inhibits β-catenin degradation [7], the dephosphorylation step is essential. In the absence of this reaction step, which means k_β_' is virtually zero in the equation above in this paragraph, more phosphorylation of β-catenin by PKA will decrease the β-catenin level. This is because, without the dephosphorylation step the influx at SS needs to equal to the sum of both out-fluxes shown in Figure 4. Increase of k_β _will decrease the β-catenin. In addition, the k_β_'/k_βdeg _ratio needs to be much smaller than 1. The latter term in the denominator, the term *k*_β_/*(1+k*_β_*'/k*_β*deg*_*) *(when k_β*deg *_and k_β _were varied due to the change of PKA activity), needs to dominate over k_13 _so that increased inhibition of degradation can enhance TCF activity. A computed result is shown in Figure 5.

To test this analysis further, computations with varied *k*_β _values, which make the term *k*_β_/*(1+k*_β_*'/k*_β*deg*_*) *smaller or larger than k_13_, were carried out as well. When k_β _was equal to or 1 order smaller than k_13_, the level of un-phosphorylated β-catenin did not change significantly when the β-catenin_+ _degradation constant, k_β*deg*_, was varied (shown in the dashed line in Figure 5), consistent with the above analysis. When k_β _was increased, the term *k*_β_/*(1+k*_β_*'/k*_β*deg*_*) *gradually dominates over k_13_. The k_β _value was increased up to 1.0. In the range of 0.5–1.0, a significant change in the un-phosphorylated β-catenin level due to varying k_β*deg *_was seen, as shown in Figure 5 (thin solid line with open circles). The TCF/β-catenin level varied by about 1 order within the range examined in Figure 5 (thin solid line with solid circles). The TCF/β-catenin complex varied less than β-catenin because of the rapid equilibrium of the reaction β-catenin + TCF ↔ β-catenin/TCF with dissociation constant 30 nM; the total concentration of TCF was 15 nM [2]. The results then account for the observed, significant change in TCF activity described above. It is noted here that non-GSK3β phosphorylation of β-catenin by PKA takes place at Ser675 of β-catenin, which is distinct from the sites of phosphorylation by GSK3β [7]. Mutation of Ser675 to alanine stabilizes β-catenin, attenuates inhibition of β-catenin degradation, and enhances TCF activity [7]. On the basis of these observations, reaction step 20 was added to the Lee-Heinrich model and is a degradation channel for β-catenin additional to those in the Lee-Heinrich model. PKA was assumed to inhibit degradation at reaction step 20 only. The above analysis of Eqs. (1) and (2) is based on these assumptions. Furthermore, to examine whether the added reactions at β-catenin*_+ _affect the added reactions at β-catenin_+ _and the level of un-phosphorylated β-catenin, computations with both added reaction parts present in the model were also carried out. Variations of both degradation rate constants in the added reaction steps, which may change the level of β-catenin significantly, showed that the computed results are similar to those obtained with each part computed separately in the model as shown in Figure 5. This is because, in the model, one key assumption is the rapid equilibration of reaction step 8, β-catenin/APC*/Axin*/GSK3β ↔ APC*/Axin*/GSK3β + β-catenin, with dissociation equilibrium constant of 120 nM [2]. Because of the low level of Axin and APC*/Axin*/GSK3β, on the 10^-4 ^and 10^-2 ^nM scales, respectively, the level of un-phosphorylated β-catenin (on the tens of nM scale) is roughly 4 and 1–2 orders higher than the APC*/Axin*/GSK3β/β-catenin complex and the downstream phosphorylated β-catenin, β-catenin*, respectively. Therefore, the presence of the added reaction steps at β-catenin*_+ _has no significant effect on the SS level of components in the added reaction step at β-catenin_+ _or the un-phosphorylated β-catenin. The added reaction steps at β-catenin_+ _alone can describe the parameter dependence of β-catenin level due to non-GSK3β phosphorylation, the succeeding degradation, and the resulting TCF activity. Finally, to verify the findings in the present computational study, it is suggested that the following be investigated in future experiments: (1) to seek the phosphatase of β-catenin_+ _and obtain its kinetic rate constant, k_β_'. (2) to obtain the kinetic constants, k_β _and k_β*deg*_, of the added reaction steps 19 and 20, respectively, associated with non-GSK3β phosphorylation of β-catenin. In the long-run, the model will be extended to include more unexamined effects caused by other components in the course of wnt signaling pathway research in order to draw a more complete picture.

## Conclusion

The present computational study gave the following results: (1) Phosphorylation of GSK3β increases the level of β-catenin in an approximately linear fashion. (2) β-catenin non-GSK3β phosphorylation is more likely to take place at free unphorphorylated β-catenin rather than GSK3β-phosphorylated β-catenin*, thus accounting for the observed enhancement of TCF activity. (3) To account for the effects of kinases such as PKA, which phosphorylates β-catenin and inhibits β-catenin degradation at the same time, the dephosphorylation step is essential. In addition, the kinetic parameters of the reactions taking place at the unphosphorylated β-catenin site need to meet the conditions described above in describing the simplified mathematical functions (1) and (2). Finally, the qualitative behaviors found in the present study should be useful for future experiments in charactering how the effects of GSK3β phosphorylation, β-catenin non-GSK3β phosphorylations, and β-catenin degradation, affect the kinetics of the wnt pathway, and can be verified in future experiments.

## List of abbreviations

GSK3: Glycogen synthase kinase 3; PKA: Protein kinase A; TCF: T-cell factor.

## Competing interests

The author declares that they have no competing interests.
